# First Presentation of Systemic Lupus Erythematosus in a 24-Year-Old Male following mRNA COVID-19 Vaccine

**DOI:** 10.1155/2022/9698138

**Published:** 2022-02-01

**Authors:** Yael Raviv, Batya Betesh-Abay, Yuliya Valdman-Grinshpoun, Liora Boehm-Cohen, Michael Kassirer, Iftach Sagy

**Affiliations:** ^1^Pulmonary Institute, Soroka University Medical Center, Beer-Sheva, Israel; ^2^Recanati School for Community Health Professions, Department of Nursing, Faculty of Health Sciences, Ben-Gurion University of the Negev, Israel and Soroka University Medical Center, Beer-Sheva, Israel; ^3^Department of Dermatology, Soroka University Medical Center, Beer Sheva, Israel; ^4^Rheumatology Unit and Clinical Research Center, Soroka University Medical Center, Beer-Sheva, Israel

## Abstract

The SARS-CoV-2 viral pandemic has had an immeasurable global impact, resulting in over 5 million deaths worldwide. Numerous vaccines were developed in an attempt to quell viral dissemination and reduce symptom severity among those infected. Systemic lupus erythematosus (SLE) is an autoimmune disease characterized by the production of antinuclear autoantibodies (ANAs) with heterogenic clinical manifestations, secondary to immune complex deposition in a multitude of organ systems. There are scarcely reported cases of SLE development following COVID-19 mRNA vaccination. We present a case of a 24-year-old male without preexisting conditions or family history of autoimmune disorders, presenting with SLE following the first dose of the SARS-CoV-2 Pfizer-BioNTech mRNA vaccine.

## 1. Introduction

Systemic lupus erythematosus (SLE) is an autoimmune disease characterized by the presence of antinuclear antibodies (ANA) and formation of immune complexes, which can directly and indirectly cause inflammatory damage to multiple organs [1]. While the prevalence of SLE is the highest among reproductive age, nonwhite women, SLE can be manifested virtually at any age, gender, or ethnic group [2]. Patients with SLE demonstrate lower cytotoxic *T* and CD4 T lymphocyte levels, both integral modulators of the humeral immune response [3, 4].

The SARS-CoV-2 viral pandemic has had an immeasurable global impact, responsible for over 5 million deaths worldwide [5]. Numerous vaccines were developed in attempt to quell viral dissemination and reduce symptom severity among those infected. The Pfizer-BioNTech BNT162b2 vaccine displayed exceptional safety and efficacy in clinical trials and in postrelease monitoring [6], and Israel was among the first countries to achieve mass immunization [7, 8]. Although Barda et al. [9] evaluated the broad range of potential adverse events documented following the BNT162b2 mRNA COVID-19 vaccine in a nationwide setting, autoimmune diseases were excluded from the analysis owing to the shortened reporting time period (42 days) deemed to be too brief for the development of autoimmune diseases [9]. Long-term (i.e., greater than one year) and rare adverse effects of the vaccine have not been fully elucidated. Studies have reviewed autoimmune dysregulations in patients with COVID-19 [10–12], and there is a paucity of studies exploring patients with prediagnosed SLE and their tolerability and susceptibility contextualized around receiving the COVID-19 mRNA vaccine [13, 14]. However, minimal reportings have described the development of SLE first erupting following the COVID-19 mRNA vaccination.

## 2. Case Report

A 24-year-old Israeli male of Ashkenazi descent, with no premorbidities or family history of autoimmune conditions, visited his family physician two days following administration of the first dose of the SARS-CoV-2 Pfizer-BioNTech mRNA vaccination complaining of a facial rash. He was prescribed calamine lotion for a suspected allergic reaction. The patient's environmental exposures included outdoor agricultural work and daily smoking. He did not drink alcohol or use illicit drugs. The patient subsequently returned to his physician ten days later due to progression and worsening of the rash, and fexofenadine trima tablet 180 mg was added.

**Figure 1:**
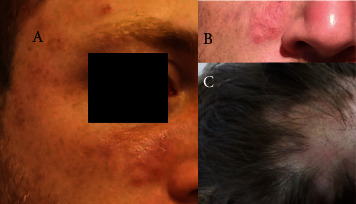
24-year-old male patient 62 days following vaccine administration. (a, b) Psoriasiform-papulosquamous plaques over the face. (c) Nonscarring hair loss over the head.

The patient presented to the Soroka University Medical Center (SUMC) 8 weeks later due to continued symptoms. At that time, the patient described multiple joint pains with morning stiffness lasting up to two hours. Dermatologist evaluation was significant for psoriasiform-papulosquamous plaques over the face, neck, and arms, nonscarring hair loss over the head (Figure 1), and bilateral wrist pain without evidence of swelling. There was normal lung sound on auscultation, without oral/nasal ulcers, xerostomia, or keratoconjunctivitis sicca. Initial laboratory results demonstrated normal range complete blood count, serum chemistries, C-reactive protein, and erythrocyte sedimentation rate. Serology testing was positive for antinuclear antibody (ANA) (1 : 160) with a speckled pattern and positive antichromatin (nucleosomal) and ribosome P antibodies (1.6 AI and >8.0 AI, respectively). Anti-double-stranded deoxyribonucleic acid, anti-Smith, SS-A, and SS-B antibodies were all negative. The C3 and C4 levels were 70 mg/dL and 21 mg/dL, respectively. Anti-cardiolipin antibodies and beta-2-glycoprotein antibodies (IgM and IgG for both), in addition to lupus anticoagulant, were negative. The direct Coombs test was also negative. Urinalysis was without casts, hematuria, or proteinuria.  Table 1 presents the chronology of symptomatic manifestations, treatment, and laboratory findings accordingly.

A diagnosis of SLE was established based on the 2019 American College of Rheumatology/European League Against Rheumatism (ACR/EULAR) classification criteria [15]. Hydroxychloroquine 200 mg BID, mometasone furoate cream 0.1%, and etoricoxib 90 mg QD as needed were initiated. The patient was instructed to avoid direct exposure to sun and to use broad-spectrum sunscreens.

The patient was recommended not to receive the second dose of the SARS-CoV-2 Pfizer-BioNTech mRNA vaccine. He was found to have COVID-19-neutralizing antibodies in the follow-up serology testing.

The patient responded well to treatment and demonstrated improvement in subsequent visits, including the disappearance of rash.

## 3. Discussion

In the case study described above, a patient exhibited an initial presentation of SLE following the mRNA COVID-19 vaccine. The exact etiology of SLE is likely multifactorial but still only incompletely elucidated. No correlation between mRNA COVID-19 vaccine administration and the imminent development of SLE has been reported. The clinical manifestations of SLE, such as nephritis, pleuritis, cardiomyopathy, and dermatitis, are mediated by antibody formation and the creation of immune complexes, ultimately causing small-vessel vasculitis. Studies have demonstrated the critical role of B-cell hyperactivity and excessive autoantibody production in SLE pathophysiology [16]. Additionally, the activation of CD4+ immune pathways has also been demonstrated in the pathophysiology of SLE. Zhao et al. demonstrated the role of microRNA in regulating DNA methylation in CD4+ T cells and its contribution to T-cell autoreactivity in SLE [17]. Additionally, the Ro/SS-A antigen is a 60 kDa protein attached to small RNAs to produce a 100 kDa complex with a nuclear localization in the skin, which can lead to adult onset of subacute cutaneous lupus erythematosus (SCLE) and neonatal lupus [18]. Hence, there is a plausible CD4+ involved in the pathogenesis of SLE that could be the biological mechanisms activated by the mRNA COVID-19 vaccine.

The notion of vaccine-induced autoimmune response has been suggested in the context of immune thrombocytopenia, autoimmune myocarditis, and autoimmune hepatitis [19–21]. While the temporal relationship may be coincidental, it is feasible that vaccine administration stimulates unregulated immunogenicity among subjects with a specific predisposition.

## 4. Conclusions

While the SARS-CoV-2 Pfizer-BioNTech mRNA vaccination has thus far demonstrated outstanding efficacy and safety, rare and long-term side effects are still not known. Further research is needed to determine if the Pfizer-BioNTech mRNA vaccine can cause untoward immune modulatory effects, including the development of autoimmune disorders such as SLE.

## Figures and Tables

**Table 1:** Chronology of clinical manifestations, laboratory results, and treatment.

	Day 0, administration of SARS-CoV-2 Pfizer-BioNTech mRNA vaccine	Day +2, visits family physician	Day +10, visits family physician	Day +33	Day +59	Day +62, presentation to the SUMC	Day +63, diagnosis made of SLE

**Clinical manifestations**		**Appearance of facial rash; nonspecific joint pain**	**Facial rash, morning stiffness, arthralgia, and fatigue**	**Progression of rash on the face, neck, and arms, nonscarring hair loss over the head, joint pain, and stiffness**		**Psoriasiform-papulosquamous plaques over the face, neck, and arms, nonscarring hair loss over the head, stress pain at the wrists without a sign of effusion, and joint pain with morning stiffness lasting up to two hours**	
**Treatment**		**Calamine topical**	**Calamine topical and fexofenadine trima tablet 180 mg**				**Hydroxychloroquine 200 mg BID; mometasone furoate cream 0.1% for topical use QD; and etoricoxib 90 mg QD as needed**
**Laboratory testing**					**Positive COVID-19-neutralizing antibodies**	**ANA:1:160, with a speckled pattern and positive anti-chromatin (nucleosomal) and ribosome P antibodies (1.6AI, >8.0AI, respectively). C3 and C4 levels: 70 mg/dL and 21 mg/dL, respectively**	

ANA: antinuclear antibody, SLE: systemic lupus erythematosus, and SUMC : Soroka University Medical Center.

## Data Availability

Data were ethically extracted from the patient's file.
